# Performance Analysis of Artificial Noise-Assisted Location-Based Beamforming in Rician Wiretap Channels

**DOI:** 10.3390/e25121626

**Published:** 2023-12-06

**Authors:** Hua Fu, Xiaoyu Zhang, Linning Peng

**Affiliations:** 1 School of Cyber Science and Engineering, Southeast University, Nanjing 210096, China; zhangxy1@js.chinamobile.com (X.Z.); pengln@seu.edu.cn (L.P.); 2 Purple Mountain Laboratories for Network and Communication Security, Nanjing 211111, China

**Keywords:** physical layer security, artificial noise, wiretap channel, location-based beamforming

## Abstract

This paper studies the performance of location-based beamforming with the presence of artificial noise (AN). Secure transmission can be achieved using the location information of the user. However, the shape of the beam depends on the number of antennas used. When the scale of the antenna array is not sufficiently large, it becomes difficult to differentiate the performance between the legitimate user and eavesdroppers nearby. In this paper, we leverage AN to minimize the area near the user with eavesdropping risk. The impact of AN is considered for both the legitimate user and the eavesdropper. Closed-form expressions are derived for the expectations of the signal to interference plus noise ratios (SINRs) and the bit error rates. Then, a secure beamforming scheme is proposed to ensure a minimum SINR requirement for the legitimate user and minimize the SINR of the eavesdropper. Numerical results show that, even with a small number of antennas, the proposed beamforming scheme can effectively degrade the performance of eavesdroppers near the legitimate user.

## 1. Introduction

Due to the broadcast nature of wireless networks, they face a variety of security threats. Passive eavesdropping is difficult to detect and prevent in wireless communication systems. Although legitimate users can engage in encrypted communication, the cost of secret key distribution is also significant. Physical layer security is promising to achieve secure transmission without key distribution. Physical layer security techniques include artificial noise, security-oriented beamforming, diversity-assisted security approaches, etc. [1,2,3]. Their security is independent of the computational capability of eavesdroppers. The multiple-input multiple-output (MIMO) technique can be used to enhance transmission security thanks to the diversity gain of beamforming.

Based on the channel state information (CSI) of the legitimate user, beamforming can be achieved towards the desired user, with a power gain higher than that of other users in different locations [4,5]. As more antennas are used at the base station, the beam becomes more concentrated. Moreover, artificial noise (AN) can be applied in the null space of the channel of the legitimate user to prevent potential eavesdropping [6,7,8,9]. Closed-form expressions for the secure transmission probability and the effective secrecy throughput are derived in [10] for a Rayleigh fading channel. The impact of the imperfect CSI is considered in [11]. A two-phase transmission scheme with AN injection is proposed in [12] to achieve zero secrecy outage probability under imperfect channel estimation. By assuming that the statistical CSI of the eavesdropper is also available at the base station [13,14,15,16], secure beamforming schemes have been proposed where the secrecy rate is maximized. Moreover, under the assumption that the CSIs of both the legitimate user and the eavesdropper are available at the base station, a secrecy capacity optimization artificial noise is proposed in [17], which is not aligned into the null space of the legitimate channel.

However, the CSI of the user is difficult to obtain by the base station in certain scenarios, such as when the user has not yet connected to the network. In addition, after the user has connected to the network, an eavesdropper can also disrupt beamforming and achieve eavesdropping through pilot contamination attacks [18,19,20]. For millimeter wave (mmWave) systems, the estimation of CSI requires a huge overhead for channel training [21].

Therefore, some works consider using alternative user information for beamforming, such as statistical CSI [13,15,16] and location information [21,22,23,24,25]. In certain scenarios, the location information of the user could be available. For example, the legitimate user is within a confidential area and dedicated networks can only be accessed within this area. We hope that users outside the confidential area cannot eavesdrop on the information of the dedicated network, because attackers can launch jamming, spoofing [26], or distributed denial-of-service attacks based on the network information. Location-based beamforming can be used to protect the dedicated networks.

The performance of location-based beamforming has been studied in [22] in Rician wiretap channels. Based on the location information of the legitimate user and the eavesdropper, the optimal location-based beamformer has been determined for the legitimate user through a grid search algorithm, which minimizes the secrecy outage probability of the system. Due to the absence of AN, eavesdroppers near legitimate users have a high secrecy outage probability when the number of antennas at the base station is small. The authors in [23] considered a wiretap system with the presence of a jammer. By assuming that the CSI of the legitimate user is known to the base station and the jammer, while the location of the eavesdropper is also available at the base station, the optimal beamformer that minimizes the secrecy outage probability has been proposed. Because the CSI between the legitimate user and the jammer is known, the AN signal is transmitted in the null space of the channel of the legitimate user. The authors of [25] studied the covert threat region of three-dimensional (3D) location-based beamforming, and the covertness performance for resisting detection from a location-unknown warden has been evaluated and compared with that of the conventional maximal ratio transmitting scheme.

In this paper, we study the performance of location-based beamforming with the assistance of AN. Based on the location information of the legitimate user, we minimize the area near the user that has eavesdropping risks. Hence, the eavesdropping performance of eavesdroppers at different locations next to the user has been studied. The impact of AN has been considered for both the legitimate user and the eavesdropper. The main contributions of this paper can be summarized as follows:

1. Based on the location information of the legitimate user and the eavesdropper, the signal to interference plus noise ratio (SINR) expressions have been derived for both the user and the eavesdropper, and the impact of AN has been considered. Close approximations of the probability density functions (PDFs) of SINRs have been proposed for Rician channels.
2. The expectations of SINRs have been derived in closed-form expressions. Moreover, the bit error rate (BER) expressions are derived using Gaussian-Laguerre (GL) approximation.
3. A quality of service (QoS)-based beamforming scheme is proposed to minimize the SINR of the eavesdropper and ensure the minimum SINR requirement of the legitimate user. Simulation results show that, when eight antennas are used at the base station, the block error rate (BLER) of eavesdroppers located 5° away from the legitimate user reaches 1.

Some works in the literature considered using ergodic secrecy rate [16,27] or secrecy outage probability [22,28] to design a secure transmission strategy. The ergodic secrecy rate refers to the difference in ergodic rate between the legitimate user and the eavesdropper. Ref. [16] proposed a power allocation algorithm for a discrete Fourier transform (DFT) beamforming matrix to maximize the ergodic secrecy rate. A deep neural network (DNN)-based secure precoding scheme is proposed in [29] to jointly design the precoder and AN signal when the channel estimation is imperfect and the channels are spatially correlated. Because the secrecy rate cannot be arbitrary values in real systems, the authors in [22,28] minimize the secrecy outage probability when a specific target secrecy rate is chosen. However, maximizing the secrecy rate does not imply that the capacity of the eavesdropper is sufficiently small. Due to the application of error correcting code (ECC) in the communication systems, an eavesdropper can successfully decode the information when the minimum SINR requirement is satisfied. To reduce the risk of eavesdropping, we consider maximizing the BLER of the eavesdropper by minimizing the SINR of the eavesdropper. QoS-based transmit beamforming has proven to be a viable and versatile approach [30]. The QoS is measured by the average SINR. Two design formulations are proposed in [30] for AN-aided secret transmit beamforming, namely a total power minimization formulation and a user’s SINR maximization formulation. The signal-to-noise ratio (SNR) outage probability criterion is proposed in [31]. In this paper, based on the error correction capability of ECC, a beamforming design scheme is proposed to minimize the SINR of an eavesdropper and maintain the minimum SINR requirement of the legitimate user. The performance of the proposed scheme is verified through simulations using Polar code [32].

Location-based beamforming can be implemented with low-cost at an analog beamforming module. Analog circuits can significantly improve the power efficiency of the device compared to digital circuits [33]. Due to the non-linear characteristics of power amplifiers, it is not suggested to adjust the amplitude of the signals for beamforming use at the radio frequency (RF) module [34]. The location-based beamformer only shifts the phase of the signal without changing its modulus, which can be considered as a phase-adjusted DFT beamformer. DFT codebook can be embedded on field-programmable analog arrays [33] with reduced power consumption. We note that when the system operates at mmWave band with an extremely large-scale antenna array (ELAA), near-field propagation needs to be considered [35]. Due to the spherical wavefront of near-field radiation, the DFT type beamforming is no longer applicable [36,37]. Secure beamforming with ELAA can be considered for our future work.

The rest of this paper is organized as follows. Section 2 introduces the system model, where the SINR expressions of the legitimate user and the eavesdropper are derived. In Section 3, the approximate PDFs of the SINRs are derived, and then the expectations of the SINRs and the BERs are deduced. In Section 4, the beamforming design scheme is proposed to minimize the SINR of the eavesdropper. The simulation results are presented in Section 5. Section 6 concludes this paper.

## 2. System Model

We consider a typical wiretap scenario, where a base station Alice and an eavesdropper Eve are equipped with uniform linear arrays (ULA) with *M* and *N* antenna elements, respectively. A legitimate user Bob is equipped with a single antenna. To facilitate the presentation of location for the users, we adopt the polar coordinate system and Alice is considered as the origin [22]. Then, the locations of Bob and Eve can be denoted as $\left(d_{ab},\theta_{b}\right)$ and $\left(d_{ae},\theta_{e}\right)$, respectively. We assume that the location of Bob is known to Alice. To investigate the impact of Eve’s location on eavesdropping performance, we assume that the location of Eve is also known to Alice. Then, an optimal beamforming scheme can be designed using AN. In the case where the location of Eve is unknown to Alice, the beamforming scheme can still be used to minimize the area with eavesdropping risk. We note that the CSIs of Bob and Eve are unknown to Alice. Based on the location information of Bob, Alice is able to transmit confidential information via a beam aiming to Bob. Moreover, Alice may transmit a jamming signal via another beam aiming to Eve. We assume that all of the channels are subject to quasi-static independent and identically distributed (i.i.d) Rician fading with different Rician K-factors, and that the K-factors are known to Alice via some a priori measurement campaigns. Hence, the channel vector from Alice to Bob, denoted as $H_{b}\in C^{1\times M}$, can be written as  

(1) $$H_{b}=\sqrt{\frac{K_{b}}{1+K_{b}}}H_{b}^{o}+\sqrt{\frac{1}{1+K_{b}}}H_{b}^{r}$$

where $K_{b}$ is the Rician K-factor of $H_{b}$, $H_{b}^{o}\in C^{1\times M}$ denotes the LOS component, and $H_{b}^{r}\in C^{1\times M}$ denotes the scattered component, the elements of which are assumed to be i.i.d. complex Gaussian random variables with zero mean and unit variance, i.e., $H_{b}^{r}∼\mathrm{CN}\left(0,I_{M}\right)$. Moreover, $H_{b}^{o}$ can be written as

(2) $$H_{b}^{o}=\left[1,e^{j\tau_{a}\cos\left(\theta_{b}\right)},...,e^{j\left(M-1\right)\tau_{a}\cos\left(\theta_{b}\right)}\right]$$

where $\tau_{a}=\frac{2\pi f_{0}\rho_{a}}{c}$, $f_{0}$ is the carrier frequency, $\rho_{a}$ is the space between two adjacent antenna elements of the ULA of Alice, and *c* is the speed of propagation of the plane wave. When $\rho_{a}$ is equal to a half wavelength, i.e., $\rho_{a}=\frac{c}{2f_{0}}$, we have $\tau_{a}=\pi$.

Likewise, the channel matrix from Alice to Eve, denoted as $H_{e}\in C^{N\times M}$, can be written as

(3) $$H_{e}=\sqrt{\frac{K_{e}}{1+K_{e}}}H_{e}^{o}+\sqrt{\frac{1}{1+K_{e}}}H_{e}^{r}$$

where $K_{e}$ is the Rician K-factor of $H_{e}$, $H_{e}^{o}\in C^{N\times M}$ denotes the LOS component, and $H_{e}^{r}\in C^{N\times M}$ denotes the scattered component with i.i.d circularly-symmetric complex Gaussian random variables with zero mean and unit variance. Moreover, $H_{e}^{o}$ can be written as

(4) $$H_{e}^{o}=h_{e}^{T}h_{ae}$$

where $h_{e}$ and $h_{ae}$ are the array responses at Eve and Alice, respectively, which can be written as

(5) $$\begin{array}{c} h_{e}=\left[1,e^{-j\tau_{e}\cos\left(\phi_{e}\right)},...,e^{-j\left(N-1\right)\tau_{e}\cos\left(\phi_{e}\right)}\right] \end{array}$$

(6) $$\begin{array}{c} h_{ae}=\left[1,e^{j\tau_{a}\cos\left(\theta_{e}\right)},...,e^{j\left(M-1\right)\tau_{a}\cos\left(\theta_{e}\right)}\right] \end{array}$$

where $\tau_{e}=\frac{2\pi f_{0}\rho_{e}}{c}$, $\rho_{e}$ is the space between two adjacent antenna elements of the ULA of Eve and $\phi_{e}$ is the direction of arrival from Alice to Eve.

The signal transmitted at Alice can be expressed as

(7) $$x_{a}=\sqrt{g_{b}}w_{b}s_{b}+\sqrt{g_{\mathrm{AN}}}w_{\mathrm{AN}}s_{\mathrm{AN}}$$

where $s_{b}$ is the normalized information signal, $s_{\mathrm{AN}}$ is the normalized jamming signal, i.e., $E\left[|s_{b}{|}^{2}\right]=E\left[|s_{\mathrm{AN}}{|}^{2}\right]=1$, $w_{b}\in C^{M\times 1}$ is the normalized beamformer for Bob, $w_{\mathrm{AN}}\in C^{M\times 1}$ is the normalized beamformer for jamming signal, i.e., ${∥w_{b}∥}^{2}={∥w_{\mathrm{AN}}∥}^{2}=1$, $g_{b}$ is the power allocated to the information signal, and $g_{\mathrm{AN}}$ is the power allocated to the jamming signal. Without loss of generality, the total transmit power of Alice is normalized to 1 and hence we have $g_{b}+g_{\mathrm{AN}}=1$.

Therefore, the signal received at Bob can be expressed as

(8) $$y_{b}=\sqrt{g_{b}}H_{b}w_{b}s_{b}+\sqrt{g_{\mathrm{AN}}}H_{b}w_{\mathrm{AN}}s_{\mathrm{AN}}+n_{b}$$

where $n_{b}$ represents the complex baseband thermal noise at Bob, such that $n_{b}∼\mathrm{CN}\left(0,\sigma_{b}^{2}\right)$.

Likewise, the signal received at Eve can be expressed as

(9) $$y_{e}=\sqrt{g_{b}}H_{e}w_{b}s_{b}+\sqrt{g_{\mathrm{AN}}}H_{e}w_{\mathrm{AN}}s_{\mathrm{AN}}+n_{e}$$

where $n_{e}$ represents the complex baseband thermal noise at Eve, such that $n_{e}∼\mathrm{CN}\left(0,\sigma_{e}^{2}I_{N}\right)$.

Then, the SINR at Bob can be written as

(10) $$\begin{array}{cc} {\mathrm{SINR}}_{b} & =\frac{g_{b}{\left|H_{b}w_{b}\right|}^{2}}{g_{\mathrm{AN}}{\left|H_{b}w_{\mathrm{AN}}\right|}^{2}+\sigma_{b}^{2}}\\ \; & =\frac{{\bar{g}}_{b}{\left|H_{b}w_{b}\right|}^{2}}{{\bar{g}}_{\mathrm{AN}}{\left|H_{b}w_{\mathrm{AN}}\right|}^{2}+1} \end{array}$$

where ${\bar{g}}_{b}=\frac{g_{b}}{\sigma_{b}^{2}}$ and ${\bar{g}}_{\mathrm{AN}}=\frac{g_{\mathrm{AN}}}{\sigma_{b}^{2}}$.

Moreover, assuming Eve applies maximum ratio combining (MRC) to combine the signals received from different antennas, the SINR at Eve can be written as

(11) $$\begin{array}{cc} {\mathrm{SINR}}_{e} & =\frac{g_{b}{∥H_{e}w_{b}∥}^{2}}{g_{\mathrm{AN}}{∥H_{e}w_{\mathrm{AN}}∥}^{2}+\sigma_{e}^{2}}\\ \; & =\frac{{\tilde{g}}_{b}{∥H_{e}w_{b}∥}^{2}}{{\tilde{g}}_{\mathrm{AN}}{∥H_{e}w_{\mathrm{AN}}∥}^{2}+1} \end{array}$$

where ${\tilde{g}}_{b}=\frac{g_{b}}{\sigma_{e}^{2}}$ and ${\tilde{g}}_{\mathrm{AN}}=\frac{g_{\mathrm{AN}}}{\sigma_{e}^{2}}$, ${∥\cdot ∥}^{2}$ denotes the square of the norm of a vector.

## 3. Performance Analysis

In this section, we first derive the PDF of the SINR for Bob and Eve, and then the expectation of the SINR and the BER performance can be deduced.

### 3.1. Distribution of the SINR

According to (1), we have

(12) $$H_{b}w_{b}=\sqrt{\frac{K_{b}}{1+K_{b}}}H_{b}^{o}w_{b}+\sqrt{\frac{1}{1+K_{b}}}H_{b}^{r}w_{b}.$$

The distribution of $|H_{b}w_{b}|$ has been analyzed in [22] for a general $w_{b}$. It has been shown that $H_{b}^{o}w_{b}$ is deterministic and $H_{b}^{r}w_{b}$ is a complex Gaussian random variable with zero mean and unit variance. Hence, $|H_{b}w_{b}|$ follows a Rician distribution with the parameters [22]:

(13) $$\begin{array}{c} {\bar{K}}_{b}=K_{b}{\left|H_{b}^{o}w_{b}\right|}^{2} \end{array}$$

(14) $$\begin{array}{c} {\bar{\Omega }}_{b}=\frac{1+K_{b}{\left|H_{b}^{o}w_{b}\right|}^{2}}{1+K_{b}} \end{array}$$

The PDF of the Rician distribution involves the modified Bessel function of the first kind, which is difficult to derive. However, the Rician distribution can be closely approximated by Nakagami distribution [38], with the parameters $m_{b}={\left({\bar{K}}_{b}+1\right)}^{2}/\left(2{\bar{K}}_{b}+1\right)$ and $\omega_{b}={\bar{\Omega }}_{b}$. Hence, ${\bar{g}}_{b}{\left|H_{b}w_{b}\right|}^{2}$ can be approximated by a gamma distribution with the PDF written as

(15) $$p_{{\bar{g}}_{b}{\left|H_{b}w_{b}\right|}^{2}}\left(x\right)=\frac{\beta_{b}^{\alpha_{b}}}{\Gamma \left(\alpha_{b}\right)}x^{\alpha_{b}-1}e^{-\beta_{b}x}$$

where $\alpha_{b}=m_{b}$, $\beta_{b}=\frac{m_{b}}{{\bar{g}}_{b}\omega_{b}}$ and Γ(·) is the gamma function.

Furthermore, we have

(16) $$H_{b}w_{\mathrm{AN}}=\sqrt{\frac{K_{b}}{1+K_{b}}}H_{b}^{o}w_{\mathrm{AN}}+\sqrt{\frac{1}{1+K_{b}}}H_{b}^{r}w_{\mathrm{AN}}.$$

Similarly, we can obtain that ${\bar{g}}_{\mathrm{AN}}{\left|H_{b}w_{\mathrm{AN}}\right|}^{2}$ can also be approximated by a gamma distribution, with the parameters $\alpha_{b,\mathrm{AN}}=m_{b,\mathrm{AN}}$ and $\beta_{b,\mathrm{AN}}=\frac{m_{b,\mathrm{AN}}}{{\bar{g}}_{\mathrm{AN}}\omega_{b\mathrm{AN}}}$ where  

(17) $$\begin{array}{c} m_{b,\mathrm{AN}}=\frac{(K_{b}|H_{b}^{o}w_{\mathrm{AN}}{{|}^{2}+1)}^{2}}{2K_{b}{\left|H_{b}^{o}w_{\mathrm{AN}}\right|}^{2}+1} \end{array}$$

(18) $$\begin{array}{c} \omega_{b,\mathrm{AN}}=\frac{1+K_{b}{\left|H_{b}^{o}w_{\mathrm{AN}}\right|}^{2}}{1+K_{b}}. \end{array}$$

Because $w_{b}$ and $w_{\mathrm{AN}}$ are independent, $|H_{b}w_{b}|$ and $|H_{b}w_{\mathrm{AN}}|$ can be considered as independent. Hence, the PDF of ${\mathrm{SINR}}_{b}$ can be obtained following a similar derivation as in [39]

(19) $$\begin{array}{cc} p_{{\mathrm{SINR}}_{b}}\left(\gamma \right) & =\int_{0}^{+\infty }\left(1+x\right)p_{{\bar{g}}_{b}{\left|H_{b}w_{b}\right|}^{2}}\left(\left(1+x\right)\gamma \right)p_{{\bar{g}}_{\mathrm{AN}}{\left|H_{b}w_{\mathrm{AN}}\right|}^{2}}\left(x\right)dx\\ \; & =\beta_{b}^{\alpha_{b}}\beta_{b,\mathrm{AN}}^{\alpha_{b,\mathrm{AN}}}\frac{\gamma^{\alpha_{b}-1}e^{-\beta_{b}\gamma }}{\Gamma \left(\alpha_{b}\right)\Gamma \left(\alpha_{b,\mathrm{AN}}\right)}\int_{0}^{+\infty }{\left(1+x\right)}^{\alpha_{b}}x^{\alpha_{b,\mathrm{AN}}-1}e^{-x\left(\beta_{b}\gamma +\beta_{b,\mathrm{AN}}\right)}dx\\ \; & =\beta_{b}^{\alpha_{b}}\beta_{b,\mathrm{AN}}^{\alpha_{b,\mathrm{AN}}}\frac{\gamma^{\alpha_{b}-1}e^{-\beta_{b}\gamma }}{\Gamma \left(\alpha_{b}\right)}U\left(\alpha_{b,\mathrm{AN}};\alpha_{b}+\alpha_{b,\mathrm{AN}}+1;\beta_{b}\gamma +\beta_{b,\mathrm{AN}}\right) \end{array}$$

where U(a;b;x) is the confluent hypergeometric function of the second kind, which is defined as [39]

(20) $$U\left(a;b;x\right)=\frac{1}{\Gamma (a)}\int_{0}^{+\infty }t^{a-1}{\left(1+t\right)}^{b-a-1}e^{-xt}dt$$

For the case $g_{\mathrm{AN}}=0$, we have $p_{{\mathrm{SNR}}_{b}}\left(\gamma \right)=p_{{\bar{g}}_{b}{\left|H_{b}w_{b}\right|}^{2}}\left(\gamma \right)$.  

For the SINR of the eavesdropper in (11), we have

(21) $${∥H_{e}w_{b}∥}^{2}=\sum_{n=1}^{N}{\left|H_{e,n}w_{b}\right|}^{2}$$

where $H_{e,n}$ is the $n^{th}$ row of $H_{e}$ which can be written as

(22) $$\begin{array}{cc} H_{e,n} & =\sqrt{\frac{K_{e}}{1+K_{e}}}H_{e,n}^{o}+\sqrt{\frac{1}{1+K_{e}}}H_{e,n}^{r}\\ \; & =\sqrt{\frac{K_{e}}{1+K_{e}}}h_{e,n}h_{ae}+\sqrt{\frac{1}{1+K_{e}}}H_{e,n}^{r} \end{array}$$

where $h_{e,n}$ is the $n^{th}$ element of $h_{e}$, i.e., $h_{e,n}=e^{-j\left(n-1\right)\tau_{e}\cos\left(\phi_{e}\right)}$ and $H_{e,n}^{r}$ is the $n^{th}$ row of $H_{e}^{r}$. Hence, $|h_{e,n}h_{ae}w_{b}\left|=\right|h_{ae}w_{b}|$, the PDF of $|H_{e,n}w_{b}{|}^{2}$ can be approximated by a gamma distribution with the parameters $\alpha_{e,n}=m_{e}$ and $\beta_{e,n}=m_{e}/\omega_{e}$ where

(23) $$\begin{array}{c} m_{e}=\frac{{\left(K_{e}{\left|h_{ae}w_{b}\right|}^{2}+1\right)}^{2}}{2K_{e}{\left|h_{ae}w_{b}\right|}^{2}+1} \end{array}$$

(24) $$\begin{array}{c} \omega_{e}=\frac{1+K_{e}{\left|h_{ae}w_{b}\right|}^{2}}{1+K_{e}}. \end{array}$$

Because the rows of $H_{e}$ are independent from each other, the PDF of term ${\tilde{g}}_{b}{∥H_{e}w_{b}∥}^{2}$ can also be approximated by a gamma distribution with the parameters $\alpha_{e}=Nm_{e}$ and $\beta_{e}=\frac{m_{e}}{{\tilde{g}}_{e}\omega_{e}}$. Moreover, the PDF of term ${\tilde{g}}_{\mathrm{AN}}{∥H_{e}w_{\mathrm{AN}}∥}^{2}$ can also be approximated by a gamma distribution with the parameters $\alpha_{e,\mathrm{AN}}=Nm_{e,\mathrm{AN}}$ and $\beta_{e,\mathrm{AN}}=\frac{m_{e,\mathrm{AN}}}{{\tilde{g}}_{e}\omega_{e,\mathrm{AN}}}$ where

(25) $$\begin{array}{c} m_{e,\mathrm{AN}}=\frac{{\left(K_{e}{\left|h_{ae}w_{\mathrm{AN}}\right|}^{2}+1\right)}^{2}}{2K_{e}{\left|h_{ae}w_{\mathrm{AN}}\right|}^{2}+1} \end{array}$$

(26) $$\begin{array}{c} \omega_{e,\mathrm{AN}}=\frac{1+K_{e}{\left|h_{ae}w_{\mathrm{AN}}\right|}^{2}}{1+K_{e}}. \end{array}$$

Therefore, the PDF of ${\mathrm{SINR}}_{e}$, denoted as $p_{{\mathrm{SINR}}_{e}}\left(\gamma \right)$, can be expressed by replacing $\alpha_{b}$, $\beta_{b}$, $\alpha_{b,\mathrm{AN}}$, and $\beta_{b,\mathrm{AN}}$ with $\alpha_{e}$, $\beta_{e}$, $\alpha_{e,\mathrm{AN}}$, and $\beta_{e,\mathrm{AN}}$ in (19).

### 3.2. Expectation of SINR

The expectation of SINR can be used to design a QoS-based beamforming scheme [30]. Using the PDF of SINR in (19), the expectation of SINR for Bob can be derived as

(27) $$\begin{array}{cc} E\left[{\mathrm{SINR}}_{b}\right] & =\int_{0}^{+\infty }\gamma p_{{\mathrm{SINR}}_{b}}\left(\gamma \right)d\gamma \\ \; & =\frac{1}{\Gamma \left(\alpha_{b}\right)}\beta_{b}^{\alpha_{b}}\beta_{b,\mathrm{AN}}^{\alpha_{b,\mathrm{AN}}}\int_{0}^{+\infty }\gamma^{\alpha_{b}}e^{-\beta_{b}\gamma }U\left(\alpha_{b,\mathrm{AN}};\alpha_{b}+\alpha_{b,\mathrm{AN}}+1;\beta_{b}\gamma +\beta_{b,\mathrm{AN}}\right)d\gamma \end{array}$$

According to [40], we have the equation

(28) $$e^{-x}U\left(a;b;x\right)=G_{1,2}^{2,0}\left(x\;|\;\begin{array}{c} a-b+1,\\ 0,1-b \end{array}\right)$$

where $G_{p,q}^{m,n}\left(x\;\begin{array}{c} a_{1},\cdots ,a_{p}\\ b_{1},\cdots ,b_{q} \end{array}\right)$ is the Meijer’s *G*-function ([41], 9.301). Then, by performing a change of variable $t=\beta_{b}\gamma +\beta_{b,\mathrm{AN}}$, a closed-form expression for expectation of SINR for Bob can be derived using [42]

(29) $$\begin{array}{cc} E\left[{\mathrm{SINR}}_{b}\right] & =\frac{1}{\Gamma \left(\alpha_{b}\right)}\beta_{b}^{\alpha_{b}}\beta_{b,\mathrm{AN}}^{\alpha_{b,\mathrm{AN}}}e^{\beta_{b,\mathrm{AN}}}\int_{0}^{+\infty }\gamma^{\alpha_{b}}G_{1,2}^{2,0}\left(\beta_{b}\gamma +\beta_{b,\mathrm{AN}}\;|\;\begin{array}{c} -\alpha_{b},\\ 0,-\alpha_{b}-\alpha_{b,\mathrm{AN}} \end{array}\right)d\gamma \\ \; & =\frac{\beta_{b,\mathrm{AN}}^{\alpha_{b,\mathrm{AN}}}}{\beta_{b}\Gamma \left(\alpha_{b}\right)}e^{\beta_{b,\mathrm{AN}}}\int_{\beta_{b,\mathrm{AN}}}^{+\infty }{\left(t-\beta_{b,\mathrm{AN}}\right)}^{\alpha_{b}}G_{1,2}^{2,0}\left(t\;|\;\begin{array}{c} -\alpha_{b},\\ 0,-\alpha_{b}-\alpha_{b,\mathrm{AN}} \end{array}\right)dt\\ \; & =\frac{\alpha_{b}}{\beta_{b}}\beta_{b,\mathrm{AN}}^{\alpha_{b}+\alpha_{b,\mathrm{AN}}+1}e^{\beta_{b,\mathrm{AN}}}G_{2,3}^{3,0}\left(\beta_{b,\mathrm{AN}}\;|\;\begin{array}{c} -\alpha_{b},0\\ -\alpha_{b}-1,0,-\alpha_{b}-\alpha_{b,\mathrm{AN}} \end{array}\right) \end{array}$$

For the case $g_{\mathrm{AN}}=0$, the expectation of SINR for Bob can be derived using ([41], 3.326), such that

(30) $$\begin{array}{cc} E\left[{\mathrm{SNR}}_{b}\right] & =\int_{0}^{+\infty }\gamma p_{{\bar{g}}_{b}{\left|H_{b}w_{b}\right|}^{2}}\left(\gamma \right)d\gamma \\ \; & =\frac{\beta_{b}^{\alpha_{b}}}{\Gamma \left(\alpha_{b}\right)}\int_{0}^{+\infty }\gamma^{\alpha_{b}}e^{-\beta_{b}\gamma }d\gamma \\ \; & =\frac{\alpha_{b}}{\beta_{b}}. \end{array}$$

Moreover, the expectation of SINR for Eve can be obtained by replacing $\alpha_{b}$, $\beta_{b}$, $\alpha_{b,\mathrm{AN}}$, and $\beta_{b,\mathrm{AN}}$ with $\alpha_{e}$, $\beta_{e}$, $\alpha_{e,\mathrm{AN}}$, and $\beta_{e,\mathrm{AN}}$ in (29) and (30).

### 3.3. BER Analysis

BER is an important metric for transmission performance. Based on the PDF of SINR in (19), the BER expression of Bob can be written as [39]

(31) $$P_{e,b}=\int_{0}^{+\infty }\frac{1}{2}\mathrm{erfc}\left(\sqrt{\gamma }\right)p_{{\mathrm{SINR}}_{b}}\left(\gamma \right)d\gamma$$

The closed-form BER expression has been derived in [39] when the parameters $\alpha_{b}$ and $\alpha_{b,\mathrm{AN}}$ are integers. Otherwise, the integration $P_{e,b}$ cannot be expressed in closed-form. In this paper, we use GL quadrature sum [43] to approximate the value of $P_{e,b}$. GL quadrature sum approximation can be expressed as

(32) $$\int_{0}^{+\infty }e^{-t}f\left(t\right)dt\approx \sum_{n=1}^{N_{GL}}w_{n}f\left(t_{n}\right)$$

where $t_{n}$ and $w_{n}$ are the abscissas and weight factors for the GL integration, which can be tabulated ([44], eq. (25.4.45)) or can be generated efficiently in software such as MATLAB R2020b. The accuracy of the GL quadrature sum increases with the number of terms $N_{GL}$ [44].

Hence, with a change of variable $\gamma =\frac{t}{\beta_{b}}$, the integration $P_{e,b}$ can be expressed as

(33) $$\begin{array}{cc} P_{e,b} & =\frac{1}{2\Gamma \left(\alpha_{b}\right)}\beta_{b}^{\alpha_{b}}\beta_{b,\mathrm{AN}}^{\alpha_{b,\mathrm{AN}}}\int_{0}^{+\infty }\mathrm{erfc}\left(\sqrt{\gamma }\right)\gamma^{\alpha_{b}-1}e^{-\beta_{b}\gamma }U\left(\alpha_{b,\mathrm{AN}};\alpha_{b}+\alpha_{b,\mathrm{AN}}+1;\beta_{b}\gamma +\beta_{b,\mathrm{AN}}\right)d\gamma \\ \; & =\frac{1}{2\Gamma \left(\alpha_{b}\right)}\beta_{b,\mathrm{AN}}^{\alpha_{b,\mathrm{AN}}}\int_{0}^{+\infty }e^{-t}t^{\alpha_{b}-1}\mathrm{erfc}\left(\sqrt{\frac{t}{\beta_{b}}}\right)U\left(\alpha_{b,\mathrm{AN}};\alpha_{b}+\alpha_{b,\mathrm{AN}}+1;t+\beta_{b,\mathrm{AN}}\right)dt \end{array}$$

The integration $P_{e,b}$ can be approximated by $P_{e,b,GL}$, which can be expressed as

(34) $$\begin{array}{cc} P_{e,b,GL}= & \frac{1}{2\Gamma \left(\alpha_{b}\right)}\beta_{b,\mathrm{AN}}^{\alpha_{b,\mathrm{AN}}}\sum_{n=1}^{N_{GL}}w_{n}t_{n}^{\alpha_{b}-1}\mathrm{erfc}\left(\sqrt{\frac{t_{n}}{\beta_{b}}}\right)U\left(\alpha_{b,\mathrm{AN}};\alpha_{b}+\alpha_{b,\mathrm{AN}}+1;t_{n}+\beta_{b,\mathrm{AN}}\right). \end{array}$$

It is shown in Section 5 that the approximation curves perfectly match numerical results for $N_{GL}=300$.

For the case $g_{\mathrm{AN}}=0$, the BER expression can be derived using [45], such that

(35) $$\begin{array}{cc} P_{e,b} & =\frac{\beta_{b}^{\alpha_{b}}}{2\Gamma \left(\alpha_{b}\right)}\int_{0}^{+\infty }\gamma^{\alpha_{b}-1}e^{-\beta_{b}\gamma }\mathrm{erfc}\left(\sqrt{\gamma }\right)d\gamma \\ \; & ={\left(1+\frac{1}{\beta_{b}}\right)}^{-\alpha_{b}}\frac{\Gamma \left(\alpha_{b}+\frac{1}{2}\right)}{2\sqrt{\pi }\Gamma \left(\alpha_{b}+1\right)}{}_{2}F_{1}\left(\alpha_{b},\frac{1}{2};\alpha_{b}+1;\frac{1}{1+\frac{1}{\beta_{b}}}\right) \end{array}$$

where ${}_{2}F_{1}\left(a,b;c;x\right)$ is the hypergeometric function ([41], Ch. 9.1).

## 4. Optimal Location-Based Beamforming

A location-based beamformer can be expressed as [22]

(36) $$w\left(\psi \right)=\frac{1}{\sqrt{M}}{\left[1,e^{-j\tau \cos\left(\psi \right)},...,e^{-j\left(M-1\right)\tau \cos\left(\psi \right)}\right]}^{T}$$

where $\psi \in \left[0,\pi \right]$ is the beamforming direction. The optimal beamformers can be denoted as $w_{b}^{*}=w\left(\psi_{b}^{*}\right)$ and $w_{\mathrm{AN}}^{*}=w\left(\psi_{\mathrm{AN}}^{*}\right)$ where $\psi_{b}^{*}$ and $\psi_{\mathrm{AN}}^{*}$ are the optimal beam directions for useful signal and AN, respectively.

In this paper, the beamforming scheme is designed based on the QoS of Bob and Eve. Because ECC has been widely used in wireless communication systems, the redundancy of code allows the receiver to correct a limited number of error bits. Hence, the receiver can successfully decode the message when the minimum SINR requirement is satisfied. On the other hand, AN needs to be strong enough at the eavesdropper side to make the BLER of the eavesdropper close to 1. When the scale of the antenna array is not sufficiently large at Alice, it is difficult to differentiate the performance between Bob and Eve nearby. In order to minimize the area near Bob with eavesdropping risk, it is necessary to increase the AN at Eve as much as possible without affecting the BLER of Bob. Hence, the beamforming design formulation ensures a minimum SINR requirement of Bob, denoted as ${\hat{\gamma }}_{b}$, while minimizing the SINR of Eve. In this case, the BLER of Eve is maximized.

We note that for each $\left(\psi_{b},\psi_{\mathrm{AN}}\right)$ pair, the resulted $E\left[{\mathrm{SINR}}_{b}\right]$ monotonically increases with $g_{b}$. Hence, when there exists values of $\left(\psi_{b},\psi_{\mathrm{AN}},g_{b}\right)$ such that the corresponding $E\left[{\mathrm{SINR}}_{b}\right]\geq {\hat{\gamma }}_{b}$, the values of $\left(\psi_{b}^{*},\psi_{\mathrm{AN}}^{*},g_{b}^{*}\right)$ can be approached through Algorithm 1.
**Algorithm 1** Algorithm to Determine $\psi_{b}^{*}$, $\psi_{\mathrm{AN}}^{*}$, $g_{b}^{*}$ for Location-based Beamforming**Require:** $\theta_{b}$, $\theta_{e}$**Ensure:** $\psi_{b}^{*}$, $\psi_{\mathrm{AN}}^{*}$, $g_{b}^{*}$1: **for** $0\leq \psi_{b},\psi_{\mathrm{AN}}\leq \pi$ with step size $\delta_{\psi }$ **do**2:    calculate $w_{b}$ and $w_{\mathrm{AN}}$ using (36).3:    **while** $0<g_{b}\leq 1$ with step size $\delta_{g}$ **do**4:        calculate $E\left[{\mathrm{SINR}}_{b}\right]$ using (29)5:        **if** $E\left[{\mathrm{SINR}}_{b}\right]\geq {\hat{\gamma }}_{b}$ **then**6:             $g_{b,tmp}=g_{b}$7:             calculate $E\left[{\mathrm{SINR}}_{e}\right]$ using (29) and the parameters of Eve8:             $\gamma_{e,tmp}=E\left[{\mathrm{SINR}}_{e}\right]$9:             **break**10:       **end if**11:   **end while**12: **end for**13: Choose $\psi_{b}^{*}$, $\psi_{\mathrm{AN}}^{*}$ that achieve the minimum $\gamma_{e,tmp}$, $g_{b}^{*}$ takes the value of the corresponding $g_{b,tmp}$.

## 5. Simulation Results

In this section, we first verify the analytical expressions derived above through simulations. Then, the performance of the proposed beamforming scheme Algorithm 1 is simulated for different $\theta_{e}$ and *M* values. The channel parameters are set as $K_{b}=10$ dB and $K_{e}=7$ dB and the SNR of the system is SNR=30 dB for both Bob and Eve.

**Figure 1:**
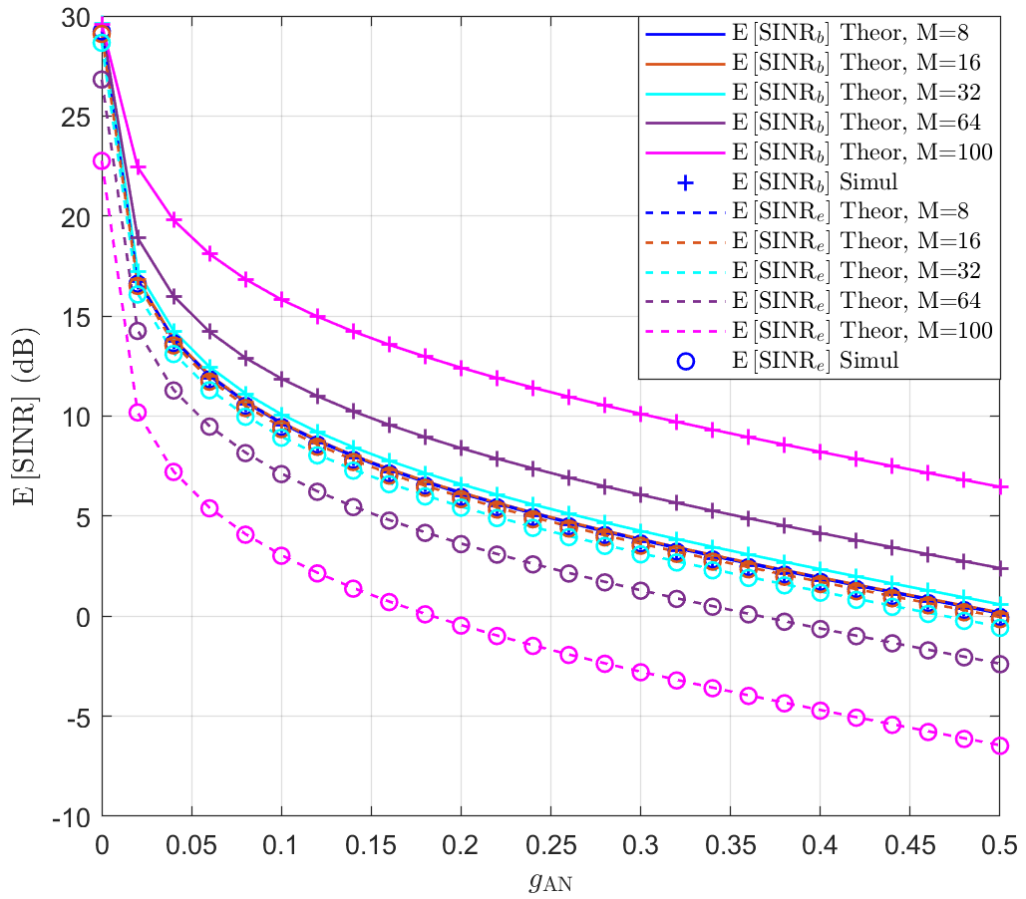
The expectation of ${\mathrm{SINR}}_{b}$ and ${\mathrm{SINR}}_{e}$ in the function of $g_{\mathrm{AN}}$ for M∈{8,16,32,64,100}.

The SINR expectation expression in (29) is tested at first assuming that $\theta_{b}=45^{\circ }$ and $\theta_{e}=46^{\circ }$. Without beamforming optimization, we align the signal beam towards Bob and the AN beam towards Eve, i.e., $w_{b}=w\left(\theta_{b}\right)$ and $w_{\mathrm{AN}}=w\left(\theta_{e}\right)$. The expectation of ${\mathrm{SINR}}_{b}$ and ${\mathrm{SINR}}_{e}$ is simulated for different *M* and $g_{\mathrm{AN}}$ values and the results are presented in Figure 1. The theoretical values of the expectation of SINR are also displayed in lines. It can be seen that the theoretical curves perfectly match the simulation results. Additionally, due to the close proximity of Bob and Eve, it can be observed that when the number of antennas is small, i.e., M≤16, the performance of Bob and Eve is almost the same, regardless of whether AN is used. This is because the beam is wide for these cases. As the number of antennas increases, the beam becomes more focused, and even without using AN, the performance of Bob and Eve becomes distinguishable. When AN is added, the performance of both Bob and Eve decreases, but the performance of Eve decreases more significantly. The simulation results show that, without beam optimization, a large number of antennas is required to differentiate the performance of Bob and Eve.

**Figure 2:**
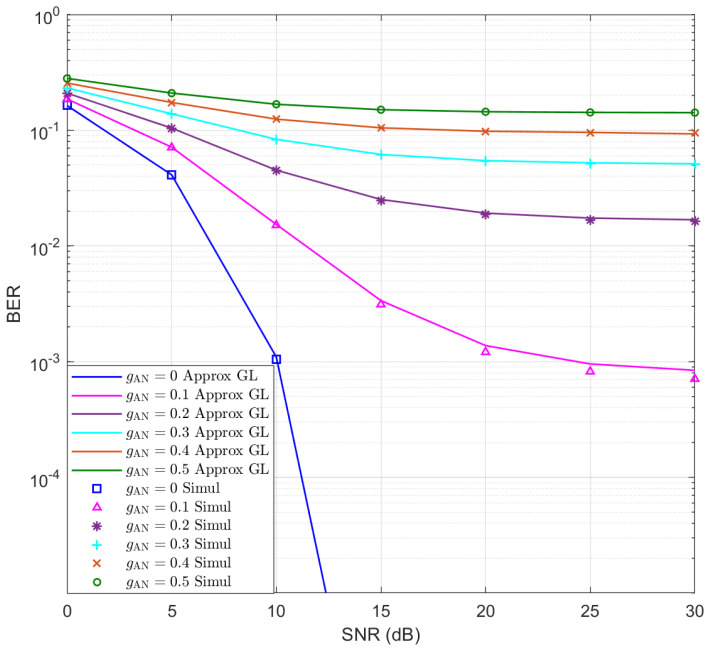
The simulated and approximate BERs of Eve for M=64.

Moreover, based on the above assumptions, the BER performance of Eve is simulated for M=64 and the results are presented in Figure 2 for different $g_{\mathrm{AN}}$ values. The approximate BERs using GL quadrature sum with $N_{GL}=300$ are presented as continuous lines, which exhibit good accuracy compared to the simulation results. Moreover, it can be seen that as $g_{\mathrm{AN}}$ increases, the BER of Eve increases rapidly.

**Figure 3:**
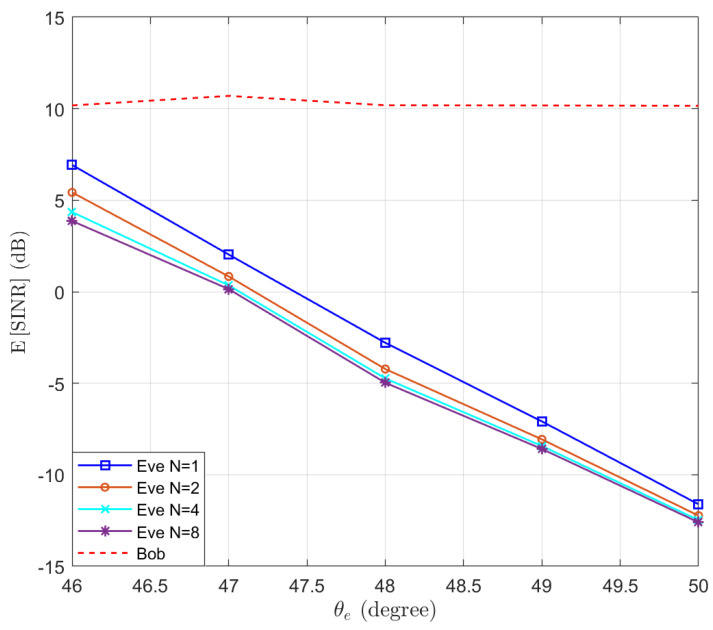
The expectation of ${\mathrm{SINR}}_{b}$ and ${\mathrm{SINR}}_{e}$ with optimal beamforming in the function of the location of Eve $\theta_{e}$.

**Figure 4:**
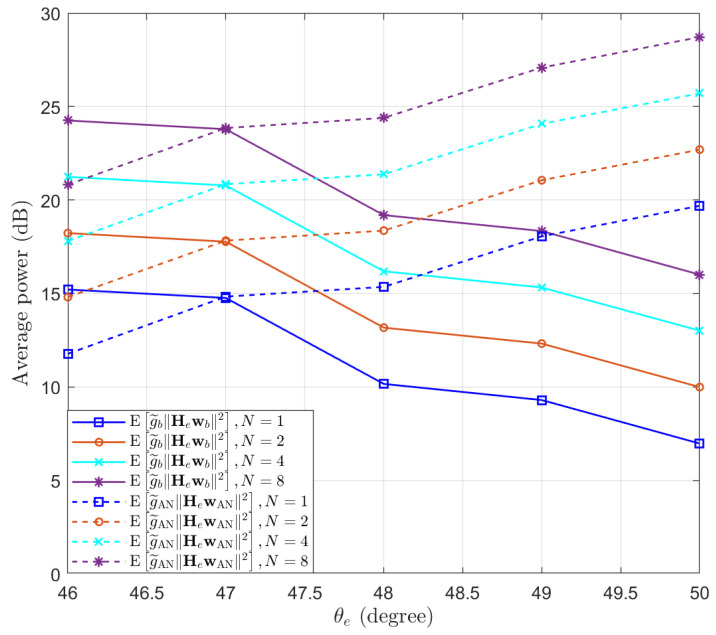
The expectation of the power of the desired signal and AN received by Eve, with different numbers of receive antennas at Eve.

Then, we test the performance of the proposed Algorithm 1 in preventing eavesdropping with a small scale of antennas. The locations of Bob and Eve remain unchanged. We set $\delta_{\psi }=1^{\circ }$, $\delta_{g}=0.01$ and ${\hat{\gamma }}_{b}=10\;\mathrm{dB}$. We test the performance of Eve at different locations, such that $\theta_{e}\in \left[46^{\circ },\cdots ,50^{\circ }\right]$. Assuming M=8 and N∈{1,2,4,8}, the expectations of ${\mathrm{SINR}}_{b}$ and ${\mathrm{SINR}}_{e}$ are simulated and the results are presented in Figure 3. We can see that the performance of Bob remains above the threshold ${\hat{\gamma }}_{b}=10$ dB, while the performance of Eve decreases when increasing the distance between Eve and Bob. Moreover, we note that when the number of antennas of Eve increases, the SINR of Eve actually decreases. This is because as the number of antennas *N* increases, the power of the desired signal and AN received by Eve both increase proportionally, resulting in a decrease in overall SINR. To clarify this point, the expectation of the power of the desired signal and AN received by Eve are displayed in Figure 4. It can be observed that as the number of receiving antennas doubles, the received power is also doubled. However, since the power of the desired signal and AN follow different distributions, the expected SINR changes with the number of receiving antennas.

**Figure 5:**
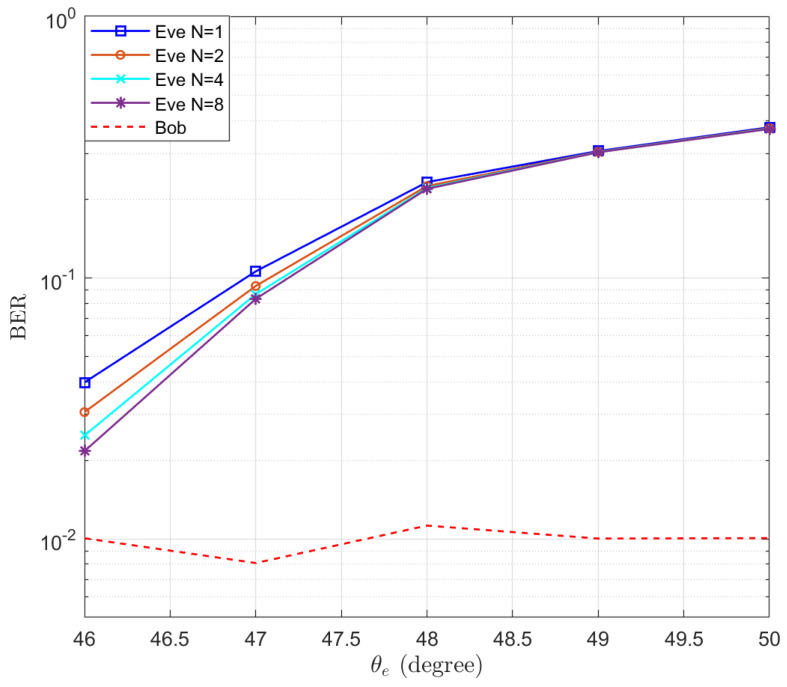
The simulated BERs of Eve in the function of the location of Eve $\theta_{e}$.

The corresponding BERs are also simulated and the results are presented in Figure 5. It is shown that the BER of Bob fluctuates around $10^{-2}$ and the BER of Eve increases when increasing the distance between Eve and Bob. When there is 5° difference between Eve and Bob, the BER of Eve reaches 0.37. Particularly, we note that the BER of the case N=8 is lower than that of the case N=1. This is in contrast to the trend exhibited by the expectation of ${\mathrm{SINR}}_{e}$ in Figure 3. This is because when a MRC receiver is used at Eve, an increase in the number of receive antennas reduces the fading of the equivalent channel. Therefore, even though the average SINR of the case N=8 is smaller than that of the case N=1, its BER is still better than that of the N=1 case, due to the diversity gain of the receive antenna array.

**Figure 6:**
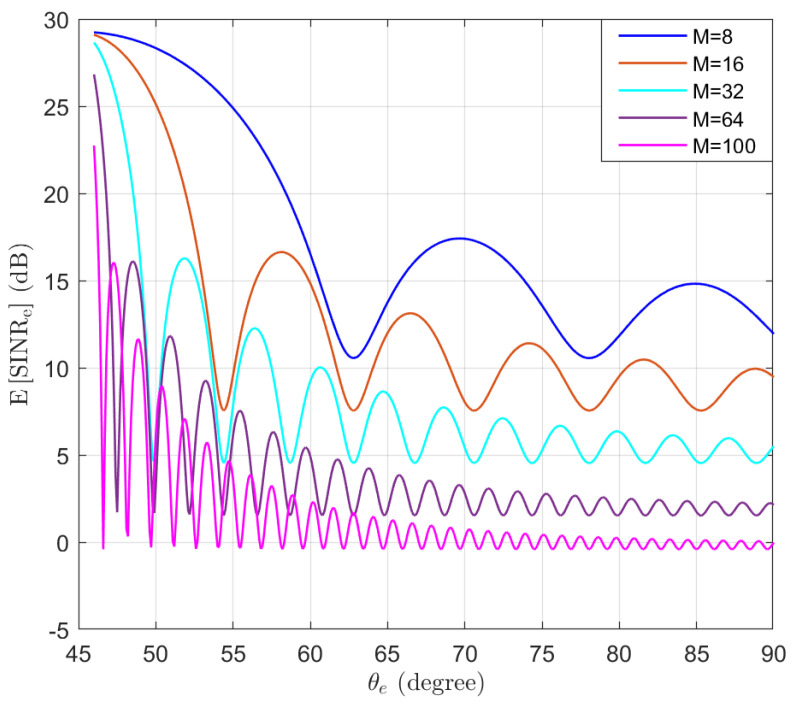
The expectation of ${\mathrm{SINR}}_{e}$ without using AN in the function of the location of Eve $\theta_{e}$.

For comparison purposes, the expectation of ${\mathrm{SINR}}_{e}$ without using AN is simulated in the function of the location of Eve $\theta_{e}$, as shown in Figure 6. The shape of the beam can be observed for different *M* values. We note that as more antennas are used, the beam becomes narrower and the sidelobes become smaller.

**Figure 7:**
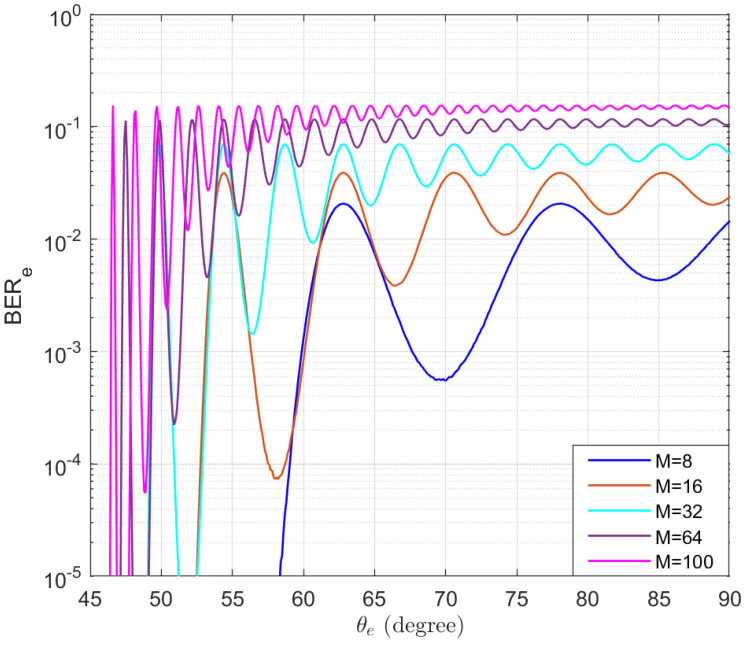
The simulated BERs of Eve without using AN in the function of the location of Eve $\theta_{e}$.

The BER performance of Eve without using AN is also simulated and the results are presented in Figure 7. It can be observed that when the number of antennas is relatively small, even if Bob and Eve are far apart, the BER of Eve remains at a low level, especially when Eve is on the peaks of the sidelobes. The results of this figure demonstrate the necessity of using AN to prevent eavesdropping.

**Figure 8:**
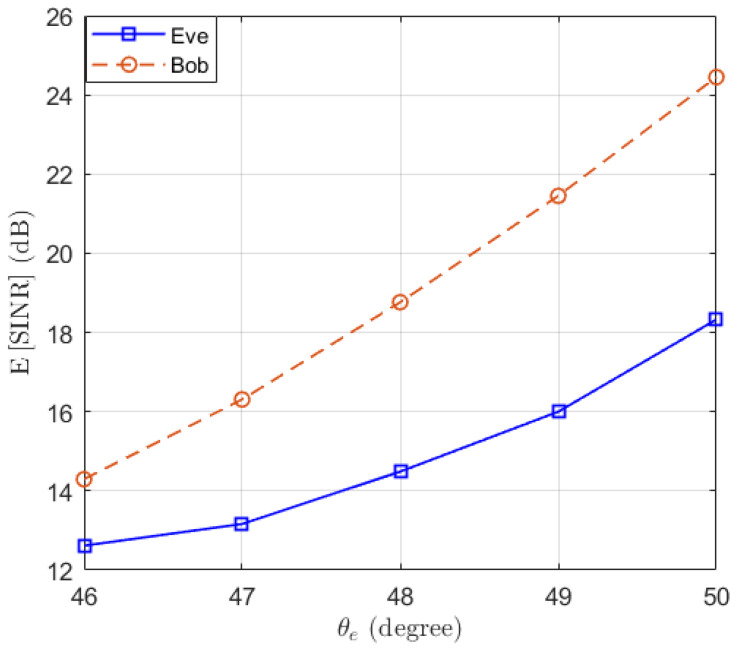
The expectations of ${\mathrm{SINR}}_{b}$ and ${\mathrm{SINR}}_{e}$ when the ergodic secrecy rate is maximized in the function of the location of Eve $\theta_{e}$.

**Figure 9:**
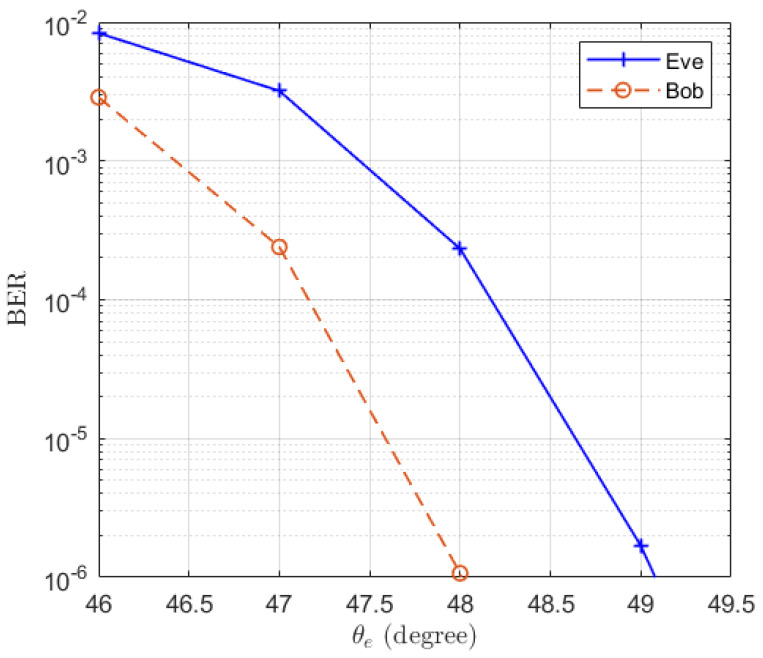
The simulated BERs when the ergodic secrecy rate is maximized in the function of the location of Eve $\theta_{e}$.

Moreover, the strategy for maximizing the ergodic secrecy rate [16] has also been simulated. The power allocation factors are optimized. The expectations of ${\mathrm{SINR}}_{b}$ and ${\mathrm{SINR}}_{e}$ are presented in Figure 8 and the BER results are shown in Figure 9. It can be observed that the difference in SINR between Bob and Eve has increased compared to Figure 3, but the BER of Eve decreases as the location of Eve becomes farther. This is because this strategy does not directly degrade the SINR of Eve.

**Figure 10:**
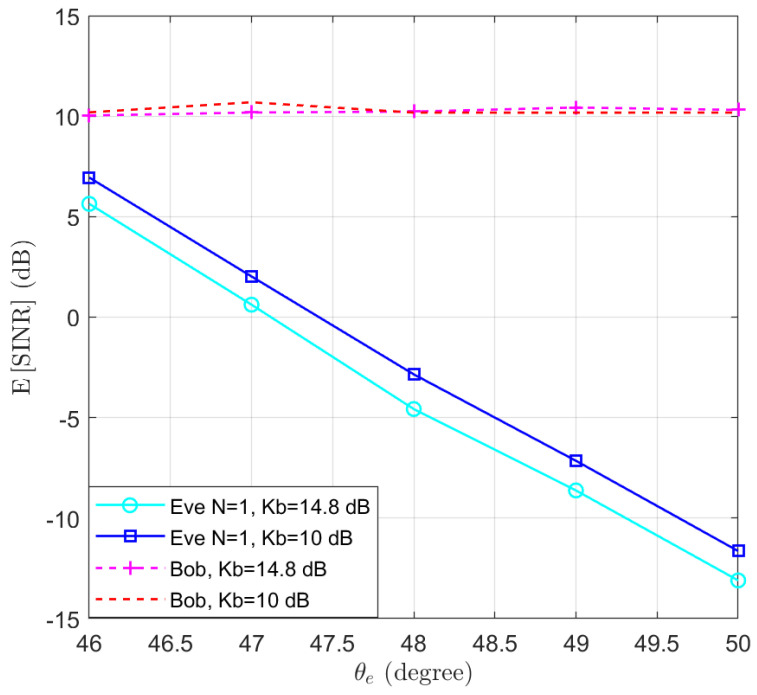
The expectation of ${\mathrm{SINR}}_{b}$ and ${\mathrm{SINR}}_{e}$ for $K_{b}=\left\{10,14.8\right\}$ dB in the function of the location of Eve $\theta_{e}$.

To clarify the impact of the Rician parameter, the case where $K_{b}=14.8$ dB is simulated to compare with the results of $K_{b}=10$ dB. The expectations of ${\mathrm{SINR}}_{b}$ and ${\mathrm{SINR}}_{e}$ are presented in Figure 10. We note that a higher $K_{b}$ value leads to a degradation in the performance of Eve. However, the trend of the eavesdropper’s SINR decreasing as the location moves away from the user remains unchanged. Hence, the proposed algorithm can be applied for LoS channels with different K-factors to degrade the SINR performance of an eavesdropper. An example of the K-factor for different scenarios can be found in ([46], Table 7.5–6).

**Figure 11:**
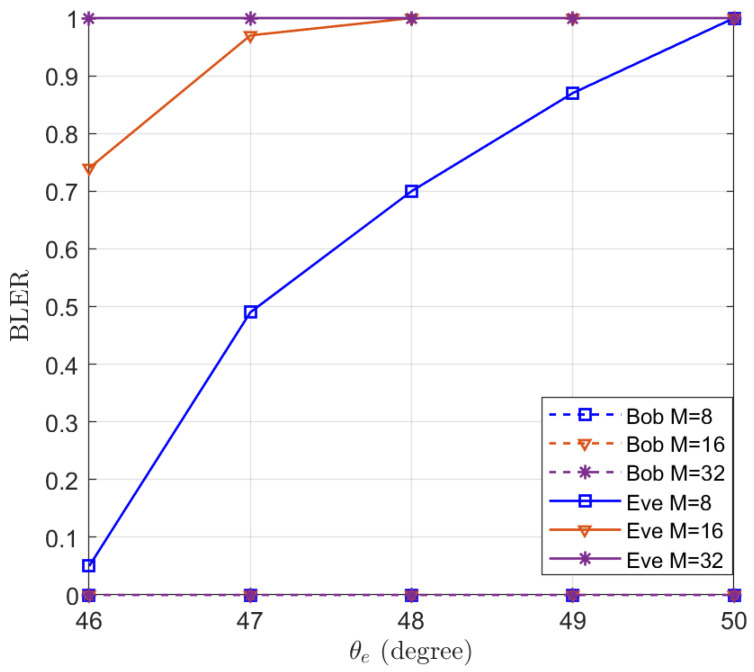
The simulated BLERs of Bob and Eve in the function of the location of Eve $\theta_{e}$.

To illustrate the effectiveness of the proposed algorithm for minimizing the QoS of the eavesdropper, the BLER of Bob and Eve are simulated in Figure 11 using Polar code as the ECC. Polar code [32] is an emerging channel coding technique for 5^*th*^ generation (5G) mobile communication systems [47]. Polar code has been adopted for enhanced mobile broadband (eMBB) control channels. We set the rate of the code R=4, ϵ=0.15, and the length of information as 128. It can be seen from Figure 11 that the BLER of Bob remains 0 and the BLER of Eve increases with $\theta_{e}$. When M=8, the BLER of Eve reaches 1 for $\theta_{e}=50^{\circ }$, which means that eavesdroppers beyond 5° cannot decode the information. When the number of antennas reaches 32, eavesdroppers beyond 1° also cannot decode the message.

In the case where the location of Eve is unknown to Alice, Alice can design beamforming schemes using the location of Eve that results in a sufficiently low BLER for Eve. In this way, the chosen location of Eve and more distant areas are protected. For the region between Bob and the chosen location of Eve, additional surveillance measures can be employed to prevent eavesdropping.

## 6. Conclusions

This work investigated the performance of AN-assisted location-based beamforming in Rician wiretap channels. Assuming that the location information of the legitimate user and the eavesdropper is available at the base station, AN is used to interfere with eavesdroppers. The influence of the AN has been considered for both the legitimate user and the eavesdropper. Closed-form PDF approximations of the SINRs are derived. Moreover, the expressions of the expectations of the SINRs and the BERs are deduced. A secure beamforming scheme is proposed to ensure a minimum SINR requirement for the legitimate user and minimize the SINR of the eavesdropper. Numerical results show that the proposed beamforming scheme can effectively degrade the performance of nearby eavesdroppers even with a small number of antennas. When the base station has eight antennas, the BLER of the eavesdropper reaches 1 when the eavesdropper is located 5° away from the legitimate user, while the BLER of the legitimate user remains 0. In the case where the location of the eavesdropper is unknown to the base station, the proposed beamforming scheme can still be used to minimize the area near the legitimate user with eavesdropping risk. When more antennas are used at the base station, the area with eavesdropping risk can be further reduced.

## Data Availability

The data presented in this study are available on request from the corresponding author.

## References

[1] Zou Y., Zhu J., Wang X., Hanzo L. (2016). A survey on wireless security: Technical challenges, recent advances, and future trends. Proc. IEEE.

[2] Wu Y., Khisti A., Xiao C., Caire G., Wong K.K., Gao X. (2018). A Survey of Physical Layer Security Techniques for 5G Wireless Networks and Challenges Ahead. IEEE J. Sel. Areas Commun..

[3] Sanenga A., Mapunda G.A., Jacob T.M.L., Marata L., Basutli B., Chuma J.M. (2020). An Overview of Key Technologies in Physical Layer Security. Entropy.

[4] Khisti A., Wornell G.W. (2010). Secure transmission with multiple antennas I: The MISOME wiretap channel. IEEE Trans. Inf. Theory.

[5] Li X., Jin S., Suraweera H.A., Hou J., Gao X. (2016). Statistical 3-D beamforming for large-scale MIMO downlink systems over Rician fading channels. IEEE Trans. Commun..

[6] Goel S., Negi R. (2008). Guaranteeing secrecy using artificial noise. IEEE Trans. Wirel. Commun..

[7] Zhang X., Zhou X., McKay M.R. (2013). On the design of artificial-noise-aided secure multi-antenna transmission in slow fading channels. IEEE Trans. Veh. Technol..

[8] Shang P., Yu W., Zhang K., Jiang X.Q., Kim S. (2019). Secrecy enhancing scheme for spatial modulation using antenna selection and artificial noise. Entropy.

[9] Joung J., Choi J., Jung B.C., Yu S. (2019). Artificial noise injection and its power loading methods for secure space-time line coded systems. Entropy.

[10] Yang N., Elkashlan M., Duong T.Q., Yuan J., Malaney R. (2016). Optimal Transmission With Artificial Noise in MISOME Wiretap Channels. IEEE Trans. Veh. Technol..

[11] Bai J., Dong T., Zhang Q., Wang S., Li N. (2020). Coordinated Beamforming and Artificial Noise in the Downlink Secure Multi-Cell MIMO Systems Under Imperfect CSI. IEEE Wireless Commun. Lett..

[12] Ta H.Q., Cao L., Oh H. (2023). Novel Noise Injection Scheme to Guarantee Zero Secrecy Outage under Imperfect CSI. Entropy.

[13] Zappone A., Lin P.H., Jorswieck E. (2016). Energy efficiency of confidential multi-antenna systems with artificial noise and statistical CSI. IEEE J. Sel. Topics Signal Process..

[14] Hu L., Wu B., Tang J., Pan F., Wen H. (2016). Outage constrained secrecy rate maximization using artificial-noise aided beamforming and cooperative jamming. Proceedings of the 2016 IEEE International Conference on Communications (ICC).

[15] Wang W., Teh K.C., Li K.H. (2016). Secrecy throughput maximization for MISO multi-eavesdropper wiretap channels. IEEE Trans. Inf. Forensics Secur..

[16] Wang W., Chen X., You L., Yi X., Gao X. (2019). Artificial noise assisted secure massive MIMO transmission exploiting statistical CSI. IEEE Commun. Lett..

[17] Gu Y., Wu Z., Yin Z., Zhang X. (2019). The Secrecy Capacity Optimization Artificial Noise: A New Type of Artificial Noise for Secure Communication in MIMO System. IEEE Access.

[18] Tugnait J.K. (2018). Pilot spoofing attack detection and countermeasure. IEEE Trans. Commun..

[19] Huang K.W., Wang H.M., Wu Y., Schober R. (2018). Pilot spoofing attack by multiple eavesdroppers. IEEE Trans. Wirel. Commun..

[20] Xiong Q., Liang Y.C., Li K.H., Gong Y., Han S. (2016). Secure transmission against pilot spoofing attack: A two-way training-based scheme. IEEE Trans. Inf. Forensics Secur..

[21] Xing Z., Wang R., Yuan X., Wu J. (2023). Location Information Assisted Beamforming Design for Reconfigurable Intelligent Surface Aided Communication Systems. IEEE Trans. Wirel. Commun..

[22] Yan S., Malaney R. (2015). Location-based beamforming for enhancing secrecy in Rician wiretap channels. IEEE Trans. Wirel. Commun..

[23] Liu C., Malaney R. (2016). Location-based beamforming and physical layer security in Rician wiretap channels. IEEE Trans. Wirel. Commun..

[24] Abdelreheem A., Mohamed E.M., Esmaiel H. (2018). Location-Based Millimeter Wave Multi-Level Beamforming Using Compressive Sensing. IEEE Commun. Lett..

[25] Lin Y., Jin L., Huang K., Zhong Z., Han Q. (2022). Covert Threat Region Analysis of 3-D Location-Based Beamforming in Rician Channel. IEEE Wirel. Commun. Lett..

[26] Lichtman M., Rao R., Marojevic V., Reed J., Jover R.P. 5G NR jamming, spoofing, and sniffing: Threat assessment and mitigation. Proceedings of the 2018 IEEE International Conference on Communications Workshops (ICC Workshops).

[27] Xu W., Li B., Tao L., Xiang W. (2021). Artificial Noise Assisted Secure Transmission for Uplink of Massive MIMO Systems. IEEE Trans. Veh. Technol..

[28] Hu D., Mu P., Zhang W., Wang W. (2020). Minimization of Secrecy Outage Probability With Artificial-Noise-Aided Beamforming for MISO Wiretap Channels. IEEE Commun. Lett..

[29] Yun S., Kang J.M., Kim I.M., Ha J. (2020). Deep Artificial Noise: Deep Learning-Based Precoding Optimization for Artificial Noise Scheme. IEEE Trans. Veh. Technol..

[30] Liao W.C., Chang T.H., Ma W.K., Chi C.Y. (2010). QoS-based transmit beamforming in the presence of eavesdroppers: An optimized artificial-noise-aided approach. IEEE Trans. Signal Process..

[31] Wang J., Han S., Xu S., Li J. (2023). SNR-Outage-Based Robust Artificial Noise-Aided Beamforming for Correlated MISO Wiretap Channels Under Gaussian Channel Uncertainties. IEEE Syst. J..

[32] Arikan E. (2009). Channel polarization: A method for constructing capacity-achieving codes for symmetric binary-input memoryless channels. IEEE Trans. Inf. Theory.

[33] Suh S., Basu A., Schlottmann C., Hasler P.E., Barry J.R. (2011). Low-power discrete Fourier transform for OFDM: A programmable analog approach. IEEE Trans. Circuits Syst..

[34] Han Y., Jin S., Zhang J., Zhang J., Wong K.K. (2018). DFT-based hybrid beamforming multiuser systems: Rate analysis and beam selection. IEEE J. Sel. Topics Signal Process..

[35] Cui M., Wu Z., Lu Y., Wei X., Dai L. (2023). Near-Field MIMO Communications for 6G: Fundamentals, Challenges, Potentials, and Future Directions. IEEE Commun. Mag..

[36] Lu H., Zeng Y. (2022). Near-Field Modeling and Performance Analysis for Multi-User Extremely Large-Scale MIMO Communication. IEEE Commun. Lett..

[37] Shen D., Dai L., Su X., Suo S. (2023). Multi-Beam Design for Near-Field Extremely Large-Scale RIS-Aided Wireless Communications. IEEE Trans. Green Commun. Netw..

[38] Goldsmith A. (2005). Wireless Communications.

[39] Aalo V., Zhang J. (2001). Performance analysis of maximal ratio combining in the presence of multiple equal-power cochannel interferers in a Nakagami fading channel. IEEE Trans. Veh. Technol..

[40] Wolfram HypergeometricU. http://functions.wolfram.com/07.33.26.0007.01.

[41] Gradshteyn I.S., Ryzhik I.M. (2007). Table of Integrals, Series, and Products.

[42] Wolfram MeijerG. http://functions.wolfram.com/07.34.21.0085.01.

[43] Atapattu S., Tellambura C., Jiang H. (2011). A mixture Gamma distribution to model the SNR of wireless channels. IEEE Trans. Wirel. Commun..

[44] Abramowitz M., Stegun I.A. (1972). Handbook of Mathematical Functions: With Formulas, Graphs, and Mathematical Tables.

[45] Shin H., Lee J.H. (2004). On the error probability of binary and M-ary signals in Nakagami-m fading channels. IEEE Trans. Commun..

[46] 3rd Generation Partnership Project (3GPP) (2019). Study on Channel Model for Frequencies from 0.5 to 100 GHz (Release 16).

[47] Dong L., Zhao H., Chen Y., Chen D., Wang T., Lu L., Zhang B., Hu L., Gu L., Li B. (2017). Introduction on IMT-2020 5G trials in China. IEEE J. Sel. Areas Commun..

